# Combining Signals for EEG-Free Arousal Detection during Home Sleep Testing: A Retrospective Study

**DOI:** 10.3390/diagnostics14182077

**Published:** 2024-09-19

**Authors:** Safa Boudabous, Juliette Millet, Emmanuel Bacry

**Affiliations:** 1 CEREMADE, CNRS-UMR 7534, Université Paris-Dauphine PSL, 75016 Paris, France; bacry@ceremade.dauphine.fr; 2 Mitral, Apneal, 75013 Paris, France; juliette.millet@apneal.ai

**Keywords:** arousal, home testing, autonomic markers, autonomic nervous system, machine learning, deep learning

## Abstract

**Introduction:** Accurately detecting arousal events during sleep is essential for evaluating sleep quality and diagnosing sleep disorders, such as sleep apnea/hypopnea syndrome. While the American Academy of Sleep Medicine guidelines associate arousal events with electroencephalogram (EEG) signal variations, EEGs are often not recorded during home sleep testing (HST) using wearable devices or smartphone applications. **Objectives:** The primary objective of this study was to explore the potential of alternatively relying on combinations of easily measurable physiological signals during HST for arousal detection where EEGs are not recorded. **Methods:** We conducted a data-driven retrospective study following an incremental device-agnostic analysis approach, where we simulated a limited-channel setting using polysomnography data and used deep learning to automate the detection task. During the analysis, we tested multiple signal combinations to evaluate their potential effectiveness. We trained and evaluated the model on the Multi-Ethnic Study of Atherosclerosis dataset. **Results:** The results demonstrated that combining multiple signals significantly improved performance compared with single-input signal models. Notably, combining thoracic effort, heart rate, and a wake/sleep indicator signal achieved competitive performance compared with the state-of-the-art DeepCAD model using electrocardiogram as input with an average precision of 61.59% and an average recall of 56.46% across the test records. **Conclusions:** This study demonstrated the potential of combining easy-to-record HST signals to characterize the autonomic markers of arousal better. It provides valuable insights to HST device designers on signals that improve EEG-free arousal detection.

## 1. Introduction

Frequent arousals during sleep are an influential marker of a pathologically disordered sleep night. Several sleep disorders are known to cause recurrent arousal outbreaks during sleep [1]. One such disorder is Obstructive Sleep Apnea/Hypopnea Syndrome (OSAHS) [2,3]. In OSAHS patients, arousals occur frequently during the recovery phase after an apnea/hypopnea event. The arousal allows the restoration of normal breathing and offsets hypoxia due to inspiratory resistance linked to upper airway narrowing. Arousals do not refer to long periods of full behavioral wakefulness often perceived during sleep. They are rather brief transient interruptions of sleep that often go unnoticed but disrupt sleep cycles significantly. The frequency of sleep arousals is often estimated during polysomnography (PSG). PSG is the standard sleep study commonly used for assessing sleep quality and diagnosing sleep disorders. It involves the patient spending a night with multiple sensors attached to him for the overnight measurement of different biological signals, including brain waves from the electroencephalogram required for arousal identification according to the American Academy of Sleep Medicine’s (AASM) recommended definition.

While the electrode placement for PSG is non-invasive and painless, patients commonly experience discomfort, stress, and difficulty falling asleep due to the multiple external wires. This may lead to frequent awakenings and hinder the study results. With the continuous development of sensing techniques, various lightweight diagnostic tools have been proposed as alternatives for PSG, including wearable devices like the Belun Sleep Ring [4], PneaVoX [5] and Clebre [6] tracheal sound systems, the neck-cuff system proposed in [7], and the Wearable Intelligent Sleep Monitor (WISM) [8], which is a device placed above the palmar thenar major muscles. Smartphone-based sleep testing solutions where no extra devices are needed have also been proposed, such as Apneal application [9], which records data from a smartphone attached to the patient’s chest. These alternative tools record fewer signals than full PSG. They mainly focus on parameters like heart rate, respiratory effort, breathing sounds, movement activity, and body position, and they often do not record EEG signals, limiting their use for arousal scoring.

Several previous studies have shown that different autonomic responses can be used as sensitive markers of transient sleep arousals, such as increases in respiratory rate, heart rate, blood pressure, and skin vasoconstriction. Studies based on auditory-simulated arousal have shown that arousal events lead to vasoconstriction [10], variations in blood pressure [11], and an increase in ventilation [12]. In [1], Catcheside et al. compared cardiovascular responses with arousal during normoxia and hypoxia. Their study results confirmed that changes in heart rate, pulse transit time, and skin blood flow were primarily related to arousal rather than hypoxia. In [13], Davies et al. also reported that arousal occurrence changes systolic blood pressure. Smith et al. [14] studied cardiovascular changes concomitant to respiratory-induced and spontaneous arousals by examining different aspects of ECG. Their findings confirmed that both types of arousal imply a shortening in time between R-waves (RR interval) and time between the start of the Q wave and the end of the T wave (QT interval) with a simultaneous more extended period between the onset of atrial depolarization and the onset of ventricular depolarization (PR interval). Another study involving apneic patients [15] found that increased sympathetic activity during sleep is due to chronic exposure to the periodic episodes of hypoxia, hypercapnia, and arousal events accompanying the recurring apneas.

Some attempts have been made to identify sleep arousal without brain signals. Rule-based algorithms have been proposed associating arousals with changes in autonomic nervous system activity, such as peaks in systolic blood pressure in [13] or drops in the peripheral arterial tonometry signal’s amplitude accompanied by increases in pulse rate in [16]. In [17], Foussier et al. used a Mahalanobis distance-based ranking algorithm and the multivariate analysis of variance method to extract the best discriminative uncorrelated heart rate variability (HRV) features for arousal detection. They applied a linear mixed model to account for inter-subject variation and emphasize the individual features’ discriminative power. Basner et al. [18] employed a Bayesian approach to calculate how likely a given heartbeat is to correspond to the start of arousal. Deep learning models have also been considered for this task. Olsen et al. [19] used a feedforward neural network trained on different HRV features. Ehrlich et al. [20] used an ensemble of fully convolutional networks to learn to detect arousal from RR interval signals extracted from an electrocardiogram, and Li et al. [21] developed a new dedicated deep learning model called DeepCAD to identify arousal using a raw single-lead ECG signal as input. The methods and models above have used only one input signal for detecting arousals, mainly relying on cardiovascular activations except in [19], where authors experienced using sleep stages with HRV features showing significant improvement in the model accuracy.

In this work, we present a retrospective study on the large Multi-Ethnic Study of Atherosclerosis (MESA) dataset that explores the potential of combining different signals easily measurable by wearable and smartphone-based diagnostic devices to improve the identification of cortical arousals without access to brain activity. We simulate a limited channel diagnostic context using PSG records using only signals that lightweight diagnostic devices commonly record or can estimate. This simulation-based approach ensures that the study is device-type agnostic. It also allows the evaluation of signal combinations that have not yet been explored, which can drive the design of new diagnostic tools. In this study, we rely on deep learning to automate the arousal detection task. The study’s results on the MESA dataset have confirmed our initial hypothesis regarding the performance improvement achieved by combining different signals. The combination of the thoracic effort signal with heart rate and a binary indicator of wake/sleep, which annotates sleep records per 30 s epochs based on wakefulness (wake epochs are periods of complete wakefulness and differ from the transient arousals that do not necessarily lead to the patient’s awakening), resulted in competitive results compared with the state-of-the-art performance in detecting arousal events during sleep without EEG signals.

## 2. Method

Different lightweight and wearable devices have been introduced to facilitate and improve home sleep testing (HST). These devices rely on different techniques and technologies for signal acquisition, resulting in variations in the quality of the signals they capture. Several studies have compared the performances of these devices in tasks such as sleep stage classification or identifying apnea/hypopnea events. However, the comparison results may be influenced by the size of the study group and its characteristics. To address this issue, we chose to simulate a limited-channel HST setting similar to HST using wearable devices and PSG signals from the Multi-Ethnic Study of Atherosclerosis (MESA), ensuring a device-agnostic study. We only included PSG signals that can be easily recorded and accessed during HST using wearable devices or smartphone applications. We also applied lenient inclusion criteria to the MESA dataset, allowing the use of signals affected by movement or sensor misplacement noises.

### 2.1. Dataset

This study is based on data from MESA [22]. MESA is a multi-center longitudinal research study, sponsored by the National Heart, Lung, and Blood Institute (NHLBI), involving asymptomatic participants aged between 45 and 84 from six communities in the United States. The study was designed to investigate factors associated with the development and progression of subclinical cardiovascular disease in an ethnically diverse population.

Between 2010–2012, 2237 participants underwent an overnight in-home PSG as part of the follow-up exams. Institutional review board approval was obtained at each study site, and written informed consent was obtained from all participants. Only 2055 from the 2237 MESA PSG records are available in the National Sleep Research Resource (NSRR) repository [23].

PSG was conducted using a 15-channel monitor (Compumedics Somte System; Compumedics Ltd., Abbotsville, Australia). Each PSG recording includes electroencephalography (EEG), bilateral electrooculograms, chin electromyography (EMG), bipolar electrocardiography (ECG), thoracic and abdominal respiratory inductance plethysmography, airflow measured by a thermocouple and nasal pressure cannula, finger pulse oximetry, bilateral limb movement piezoelectric sensors, and a position sensor. The PSG recordings also provide a heart rate signal derived from the ECG and a snore signal measured as vibrations related to breathing at the nasal pressure cannula level. PSG signals from the MESA study are listed in Table A1 in Appendix A.1.

Certified scorers manually scored the PSG records from MESA for sleep stages and arousal events. Each record is reviewed on an epoch-by-epoch basis. Each epoch is assigned a sleep stage, and EEG change periods that meet the arousal criteria are marked. The scoring is performed according to the AASM guidelines. Additional rules are provided in the MESA Sleep Reading Center (SRC) manual of operations and scoring rules (https://sleepdata.org/datasets/mesa/files/m/browser/documentation/MESA_Sleep_Polysomnography_Scoring_Manual.pdf (last accessed on 30 August 2024)). Regarding the sleep stage scoring, the rules mainly help to guide score assignment in sleep stage transition epochs and during an arousal event. In connection with scoring arousal, the SRC scoring rules offer useful guidance for distinguishing arousal events from artifacts caused by movement or isolated bursts of delta waves. They also provide advice on scoring arousal during rapid eye movement periods. To minimize inter-scorer scoring differences, MESA scorers participated in rule-based training and regularly took part in reliability exercises, which included re-scoring a standard set of 20 MESA records.

The SRC manual presents the results of scoring reliability assessment over a set of MESA records. The results exhibit the excellent quality of wake/sleep scoring, estimating the average difference in total sleep time to be 7 min and the inter-scorer correlation for total sleep time estimation to be between 0.96 and 1. Inter- and intra-scorer reliability for arousal scoring were also evaluated. The intra-class correlation coefficients for the arousal index ranges from 0.84 to 0.99, indicating strong consistency in scoring.

**Figure A1:**
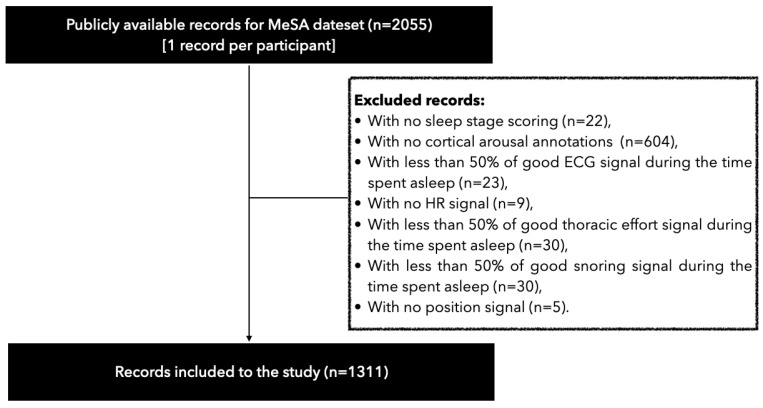
Study diagram. The flowchart shows the exclusion criteria and sample size of the final analysis.

Out of the 2055 records available, 1311 records were included for analysis. Of the discarded records (604 records), 81% lacked manual annotation for arousal, which is essential for model training and evaluation. The rest of the discarded records were mainly due to instances where at least one of the selected signals was of poor quality, defined as missing or noisy for more than 50% of sleep time. The flowchart of the exclusion criteria is depicted in Figure A1 in Appendix A.2.

### 2.2. Model Architecture

In this study, we use the same neural network architecture as the DeepCAD model. The DeepCAD model, introduced in [21], is designed to automatically identify cortical arousals using a single-lead ECG signal as its input. The model learns a non-linear function through a neural network that assigns an arousal probability to each second of an ECG signal it processes. Compared with other methods that rely on cardiac features for arousal detection, the DeepCAD model has demonstrated leading performance, as highlighted in [20].

**Figure 1:**
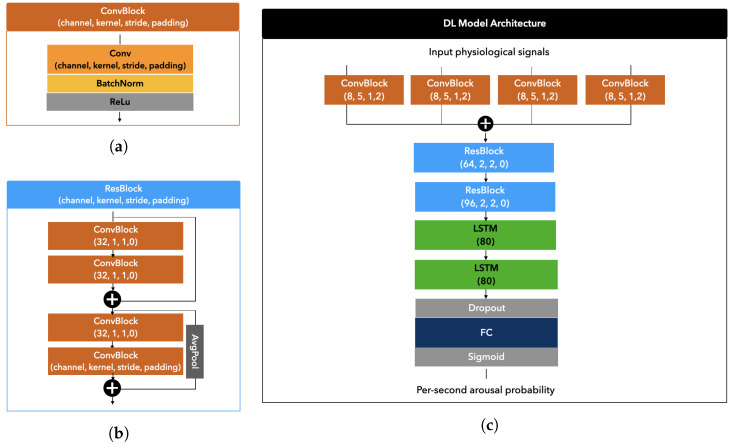
DL model architecture. We show in (**a**) the structure of the convolutional block composing both the inception and residual blocks, in (**b**) the structure of a residual block, and in (**c**) the final model architecture composed of an inception block, two residual blocks, two LSTM layers, and a final fully connected layer with a sigmoid activation.

The architecture of the DeepCAD model comprises an inception block consisting of four parallel convolutional blocks with varying reception fields, followed by residual blocks to downsample the input to 1 Hz. As shown in Figure 1b, each residual block comprises two components, each containing two convolutional blocks and corresponding skip connections. In the second component, a downsampling stride of two is employed. The output of the last residual block is fed into two Long Short-Term Memory (LSTM) layers. LSTM [24] is a type of recurrent neural network (RNN) that addresses the vanishing gradient problem of traditional RNNs and provides extended short-term memory to the network. Each LSTM unit includes a cell to remember values across multiple time steps, an input gate, an output gate, and a forget gate that all regulate the flow of information in and out of the cell. The last LSTM layer is followed by a fully connected layer with a sigmoid activation function to produce arousal probabilities.

The inception and residual blocks are based on a similar convolutional structure shown in Figure 1a, which includes a convolutional layer followed by batch normalization and ReLU activation.

In order to adapt to the 4 Hz resolution of the input signals, we use two residual blocks instead of 8 in the DeepCAD model [21]. Moreover, we adjust the kernel sizes of convolutional layers for both the inception block and the residual blocks. We consider larger reception fields for the inception’s convolutional layers by fixing kernel sizes to 5, 33, 65, and 129. We set kernel sizes to 1, 2, 7, and 2 for residual blocks.

We depict the deep learning (DL) model architecture in Figure 1c.

### 2.3. Selected Input Signals

To simulate a limited-channel HST setting, we only consider signals commonly recorded and made available during HST using wearable devices or smartphone applications. The signals we are considering consist of the heart rate, the thoracic effort, the snoring signal, the position signal, and the binary signals of wake/sleep and position change. The thoracic effort, snoring, and position signals are directly acquired with sensors during PSG. In the MESA study, standard PSG sensors were used for acquisition: a respiratory inductance plethysmograph (RIP) thoracic belt for the thoracic effort signal, a built-in movement detector for the position, and a nasal cannula for snoring. Regarding the heart rate, wake/sleep, and position change, those signals are derived from recorded PSG signals: the heart rate signal is derived from the raw ECG signal, the position change signal is a binary signal triggered to 1 when a position change is detected on the position signal, and the wake/sleep signal is extracted from the hypnogram and takes the value of 0 or 1 based on whether the patient is awake or asleep. It is important to note that the “Wake” epochs refer to periods of complete wakefulness observed in the hypnogram, which differ from arousals that may not necessarily lead to the patient’s awakening.

We point out that the signals selected from PSG can already be captured or derived by most lightweight wearable home sleep testing (HST) devices and smartphone-based solutions. These solutions use different acquisition technologies and often depend on specific algorithms and methods to extract and reconstruct each signal. For example, heart rate can be estimated using a seismocardiogram from an accelerometer placed on the patient’s chest (seismocardiography (SCG) is used by Apneal [9] to estimate the heart rate and reconstruct the thoracic effort signal). A photoplethysmogram or a tracheal sound signal can also be used to estimate the heart rate. Additionally, accelerometer and gyroscope data can be used to collect information about the patient’s position and wake/sleep stages and reconstruct the thoracic effort signal. The accuracy of measured and derived signals using a specific HST device largely depends on the device sensing technique and the defined method for signal processing. It is important to note that signals from lightweight HST devices are not always less accurate. For instance, high-quality audio recordings can better estimate the likelihood of snoring instead of relying solely on the vibration signal from the cannula.

**Figure 2:**
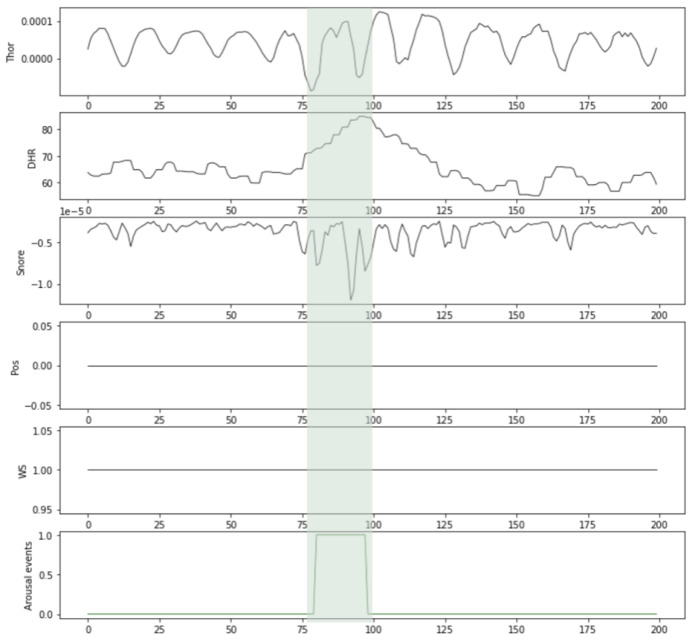
Illustrative example of measurements of selected signal (Thor, DHR, Snore, WS, Pos) around an arousal event. The green shadows indicate the manually scored arousal event.

Table 1 summarizes the selected physiological signals and their sources in the MESA dataset. An example of measurements of selected signals around a given annotated arousal event extracted from one MESA participant’s recording is illustrated in Figure 2.

The selected signals are considered as candidate input signals to the detection model. An incremental analysis approach is defined to assess each signal’s impact and identify the signal combinations that improve arousal detection without EEG signals.

### 2.4. Incremental Analysis Approach

We follow an analysis process to determine which combination of biological input signals enhances the model’s ability to detect arousals. For doing so, we use a greedy incremental approach.

**Round I:** During the first round, we train three learning models: the first using the heart rate signal as input, the second using thoracic effort, and the third using the snoring signal as input. Let us note that, in the first round, we omit the body position and wake/sleep signals listed in Table 1, since they do not make sense to be used alone for our task. We then compare the performances of these models and select, for the next round, the input signal corresponding to the model that performs best.

**Round II:** In the second round, we build pairs of input signals by adding to the previously selected signal any signal (different from the previously selected signal) listed in Table 1. We train a model for each of the pairs, and we evaluate its performance. We then select for the next round the pair of input signals that corresponds to the best-performing model.

**Successive Rounds:** In the next rounds, we follow recursively the same process by adding, at each round, a new input signal to the combination selected at the previous round. We then select, for the next round, the new combination of signals corresponding to the best-performing model.

**Figure 3:**
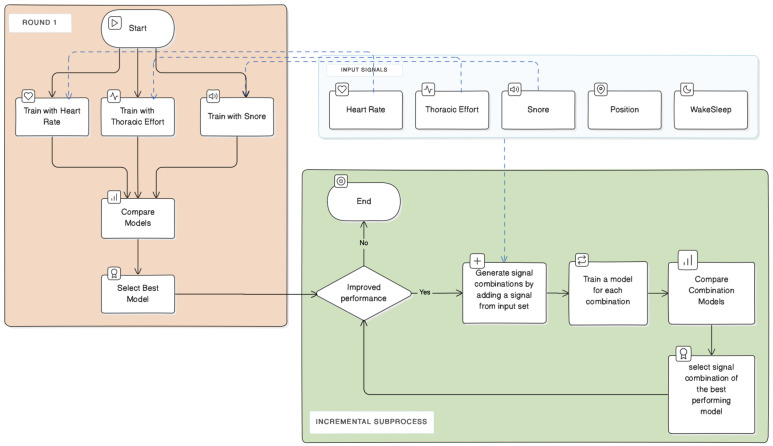
The flowchart of the incremental analysis approach. It illustrates the first round, where models trained using a single-input signal are evaluated, and the incremental subprocess, where the performance of models trained in combinations of input signals is evaluated to identify the best-performing one. In this subprocess, the input combinations are formed by adding a single signal from the list of input signal candidates to the best combination from the previous round.

We illustrate the incremental approach with a flowchart in Figure 3. We differentiate between the initial round and the incremental subprocess, including all the subsequent rounds. Each round of the incremental subprocess involves training models using input signal combinations generated by adding a signal to the input of the best model from the preceding round, evaluating their performance, and assessing for improvement.

### 2.5. Experiment

#### 2.5.1. Preprocessing

In this study, we aimed to minimize the amount of data preprocessing. First, all selected signals from PSG were resampled at 4 Hz. Then, we standardized each signal by removing the median and dividing each sample by the interquartile range. No filtering was applied to the signals to deal with noise. We only corrected outliers in the heart rate signal to address errors caused by R peak misdetection on the ECG signal.

The decision not to apply filtering is primarily due to using a deep architecture for the learned model. The model’s robustness to noise is mainly credited to the convolutional layers. These layers allow for extracting essential and relevant features from input signals through filters and moving windows and effectively skip noise and irrelevant information. It is worth noting that experiments involving filtering on some input signals (e.g., thoracic effort signal) were conducted, and they showed no significant difference in the model performance. The results of those experiments were omitted to keep the Results section clear and smooth.

#### 2.5.2. Training

The selected 1311 records from the MESA dataset were split for model training, validation, and evaluation, resulting in 952 records for training, 104 for validation, and 255 for testing. Table A2 in Appendix A.1 shows the characteristics of data from each set.

Similar to [21], we use cross-entropy loss as the loss function, and we train the models on batches of size 30 using truncated backpropagation through time [25] with a depth of 90 and an Adam optimizer $(\beta_{1}=0.9,$ $\beta_{2}=0.999,$ λ = $1\times 10^{-5}$) [26]. We also initialize the learning rate to $1\times 10^{-4}$ and reduce it by a factor of 10 when the performance stops improving for four consecutive epochs. We train the model for 30 epochs and select the model with the best area under precision–recall on the validation set.

#### 2.5.3. Postprocessing

The deep learning model takes a sequence of selected signals as input. After processing and downsampling to 1 Hz, it outputs a vector of per-second likelihoods of arousal event occurrence. These outputs result from applying a sigmoid activation function to the output of the final linear prediction layer. As the values produced by the sigmoid function range between 0 and 1, they can be intuitively interpreted as the likelihood of an event occurring at a specific time interval. The closer the output is to 1, the more likely it is that an arousal occurred. The continuous output values of the sigmoid function are thresholded to determine class labels for the binary arousal/no arousal classification task. A decision threshold is defined so that the label is set to 1 if the arousal probability is above a specified decision threshold and 0 otherwise. Arousal events are then defined as a series of continuous positive labels.

We optimize the decision threshold value to maximize the event-based F1-score on the validation set. To do this, we start by selecting three threshold values, each spaced 0.02 apart, centered on the threshold that improves the pointwise F1 score. We then adjust these values, either higher or lower, based on the order of the event-based F1 scores obtained. If the central value maximizes the event-based F1 score, we decrease the minimum and maximum values. The process is repeated until the difference between the successive values is 0.01, and the central value is the one that maximizes the event-based F1-score.

The model’s output is further postprocessed to better comply with the AASM guidelines and to smooth the model predictions. A two-step postprocessing is applied. A first merging step ensures that two arousal events are separated by at least 5 s. This 5 s gap is used instead of the 10 s gap recommended by the AASM guidelines to account for up to 3 s of uncertainty in arousal onset and end detection. The second postprocessing step discards arousals lasting less than 3 s to meet the AASM’s recommended minimum arousal duration. Table A3 in Appendix A.4 details the number and percentage of detected arousals that were combined and those that were discarded for each trained model.

### 2.6. Evaluation Metrics

To evaluate the model’s performance, we only consider the events annotated during sleep and discard any events annotated or detected during periods of stable wakefulness. This makes sense since arousals only refer to brief shifts to wakefulness from sleep. Moreover, only arousals during sleeping time are included on arousal index calculation. In our study, we used the hypnogram provided in the MESA dataset to remove wake periods from our analysis. However, it is worth noting that there exist several EEG-free sleep–wake classifiers that could be efficient enough to replace the use of a hypnogram.

We evaluate the results in three ways: pointwise, event-based, and recordwise. Unlike the recordwise evaluation, the two former evaluations are global and patient-agnostic. Point-wise evaluation enables precise assessment of the model’s performance at a resolution of one s. This means that the accuracy of the output label is evaluated at each time bin. On the other hand, event-based evaluation is more focused on assessing the model’s ability to detect arousal events as they occur and accurately identify autonomic activations related to them. Finally, record-based evaluation complements the previous global evaluation by taking into account inter-patient variability. In this type of evaluation, the stability model’s performance is assessed.

#### 2.6.1. Point-Wise Evaluation

For calculating pointwise metrics, we first concatenate test set records as one output sequence and then compare it with the sequence of reference ground truth annotations. We classify each sequence point based on the presence or no presence of an arousal event and whether it was detected.

We evaluate the model performance based on precision, recall, and F1-score metrics. We further calculate the area under the precision–recall curve (AUPRC) and the area under the receiver operating characteristic curve (AUROC).

#### 2.6.2. Event-Based Evaluation

For event-based evaluation, we consider a lenient definition of true detections based on the overlap rate between the detected and the reference events. Thus, a detection is considered true positive if it covers enough at least one ground truth arousal (i.e., there is sufficient overlapping between a certain ground truth arousal event and the detected one).

Let us denote by $\Omega_{min}$ the required minimum proportion of the ground truth event that should be covered by the model output and by $Match(G_{i},D_{j})$ the function set to 1 if the overlap criterion is satisfied between $G_{i}$ and $D_{j}$.

(1) $$Match\left(G_{i},D_{j}\right)=\left\{\begin{array}{cc} 1 & \frac{|G_{i}\cap D_{j}|}{|G_{i}|}>\Omega_{min}\\ 0 & otherwise \end{array}\right.$$

As for pointwise evaluation, we use precision, recall, and F1-score metrics. In this context, the recall metric allows for measuring the ability of the model to correctly identify true arousal events while the precision allows for measuring the accuracy of the detections made by the model. In this study, the required minimum overlap rate $\Omega_{min}$ is set to 0.5.

#### 2.6.3. Record-Wise Evaluation

In the recordwise evaluation, we compute event-based recall, precision, and F1-score separately for each test record (i.e., each patient). We analyze the distributions of recall, precision, and F1-score to assess the model performance stability. We use the bootstrapping paired *t*-test [27] with the Holm–Bonferroni multiple comparisons method [28] for significance testing when comparing the results using different input combinations. The bootstrap approach was chosen due to its lack of strict assumptions about the underlying distribution of metrics’ output, as it does not presume normality or equality of variances. Instead, it mainly relies on the assumption that the empirical distribution from the data accurately reflects the actual population characteristics. The bootstrapping paired *t*-test involves repeatedly resampling the paired differences with replacement to create a bootstrap distribution of t-statistics, which is then used to calculate a *p*-value as the proportion of bootstrap t-statistics more extreme than the original t-statistic.

When performance is compared with a state of the art, we additionally compute the arousal index (ArI) for each record as the average number of arousal events per hour of sleep. We compare the ArI based on detected events with the one using the ground truth arousal events using the Pearson correlation. We also analyze the difference between the two indexes by a Bland–Altman plot.

## 3. Results

Our analysis process results in the evaluation of 16 input data combinations. The combinations include three training settings using a single signal as input, five using two signals, six using three signals, one using four signals, and one using all available signals.

### 3.1. Round I: Model Trained with a Single Input Signal

In the first round, we train the model using a single signal from HR, Thor, and Snore to detect arousals. We show the obtained results in terms of events-based, pointwise, and recordwise evaluation in Table 2.

The results presented in Table 2 indicate that the model trained using the Thor signal as input achieved the best performance. This model achieves a 16% improvement in the pointwise F1-score and a 15% improvement in the event-based F1-score. The improvements in F1 scores result from both higher precision and recall scores. We also obtain higher AUPRC and AUROC scores when using the Thor signal.

**Figure 4:**
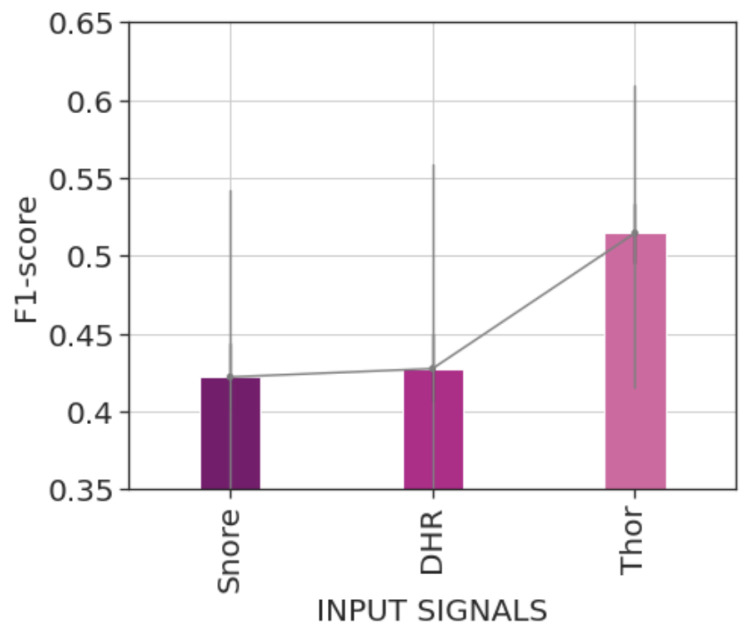
Bar plot of the average recordwise event-based F1-scores of the different trained models during the first experiment round. Error bars represent 50% percentile intervals. The model trained using the Thor signal achieves a significantly better score, reaching an average score exceeding 50%.

Figure 4 represents the average recordwise F1-scores of the trained models. We can observe that the model trained using the Thor signal significantly outperformed those trained using HR or Snore signals. As detailed in Table 2, the Thor signal model obtains a mean recordwise F1-score of 51.45% compared with a mean recordwise F1-score of around 42% by the HR or the Snore signal. The statistical significance of these results was tested using the bootstrapping test of Berg-Kirkpatrick et al. [27], as explained in Section 2.6.3.

In Table 3, we use relative confusion matrices to compare the model performance to detect arousal events using the Thor signal with its performance using the DHR or the Snore signal. Relative confusion matrices represent the counts of true events that both models commonly detect, events detected only by the first model, events detected only by the second model, and events not detected by either of the models. By focusing on the off-diagonal values, we conclude that about 18% of arousals were detected only when the model used the Thor signal as input. However, about 10% of true events were missed when using the Thor signal, while they were correctly identified using the other signals. Hence, combining the Thor signal with other physiological signals such as DHR or Snore may increase the arousal detection rate. This is what we investigate in the next round.

### 3.2. Round II: Model Trained with Two Input Signals

In this second round, we combine the Thor signal with one of the other signals at our disposal (DHR, Pos, Pos chg, Snore, and W/S signals). The obtained evaluation results are summarized in Table 4.

The results show that including one signal from DHR, Snore, or W/S to the model inputs enhances both event-based and pointwise F1-scores. Event-based and pointwise F1-scores were 56.29% and 51.22% when using only the Thor signal (cf. Table 2). Among those combinations, the one combining the Thor signal with the DHR signal achieved the highest improvement in the event-based F1-score of approximately 3.7% when compared with the model trained using only the Thor signal (cf. Table 2). Meanwhile, the model trained with the Thor and W/S signals resulted in the best improvement in pointwise AUROC, AUPRC, and F1-scores. Combining the Thor signal with position information (Pos or Pos_chg) significantly improves recall scores but results in lower precision due to more falsely detected events.

**Figure 5:**
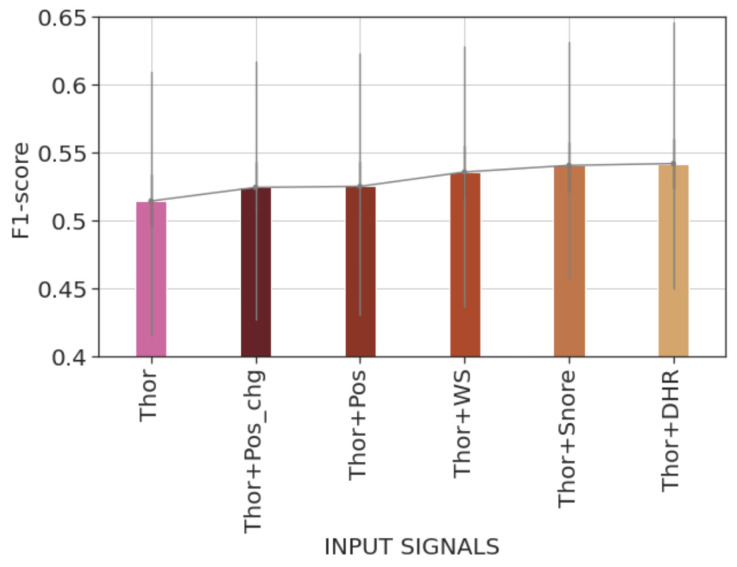
Bar plot of the average recordwise event-based F1-scores of the different trained models during the second experiment round. Error bars represent 50% percentile intervals. Combining the Thor signal with another from Pos, Pos_chg, WS, Snore, or DHR signals yields higher F1-scores. The highest score is obtained by training the model using Thor and DHR signals.

Recordwise evaluation results indicate that using a combination of signals (DHR, Snore, or W/S) along with the Thor signal provides significantly better F1-scores on test records when compared with the model using only the Thor signal (cf. Table 2). They show a statistically significant improvement in the recall score but no significant difference in the precision score over the test records. The bar plot in Figure 5 illustrates the average recordwise F1-scores of models trained during the second round of experiments. Additionally, the average F1-score of the model trained using only the Thor signal is provided for comparison. Upon comparison, we state that the model trained with the Thor and DHR signals achieves the highest average F1-score.

### 3.3. Round III: Model Trained with Three Input Signals

In the third round, we examine every possible combination of three input signals, which included the Thor signal and at least one of the DHR, Snore, or W/S signals.

We compare the results obtained from these combinations with the results of the following combinations from the previous round: Thor+DHR, Thor+Snore, and Thor+W/S (summarized in Table 4). We note further improvements in terms of event-based F1-score, especially when training the model with Thor, W/S, and DHR signals as input or when considering Thor, DHR, and Snore signals. The first combination enhances the event detection precision by at least 8% compared with previous round results (cf. Table 4). Additionally, it provides the best result in terms of pointwise AUPRC, AUROC, and F1-score while maintaining a balanced trade-off between precision and recall. On the other hand, the combination of Thor, Snore, and DHR signals gives a balanced trade-off between event-based precision and recall of around 59%. However, it has significantly higher recall (58.94%) than precision (47.21%) in pointwise evaluation.

**Figure 6:**
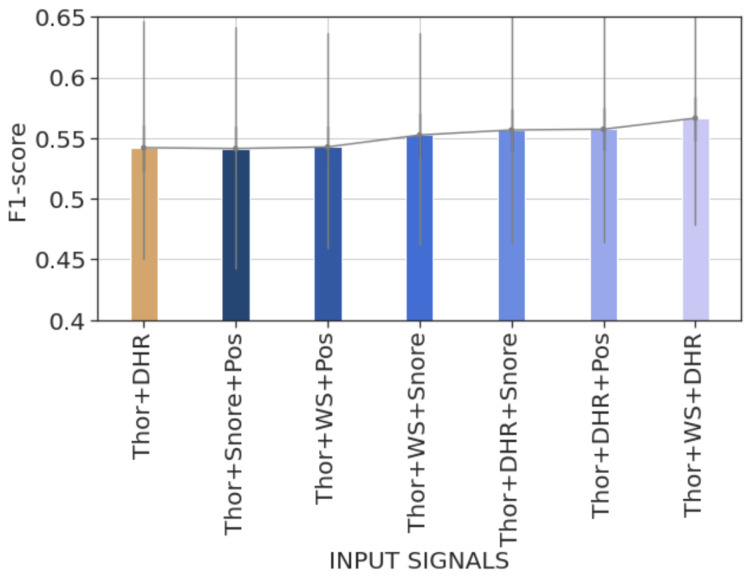
Bar plot of the average recordwise event-based F1-scores of the trained models during the third experiment round using combinations of three signals as input. Error bars represent 50% percentile intervals. The combination of Thor, WS, and DHR signals results in the best model performance in terms of F1-score among all the tested combinations.

Recordwise evaluation provides the same conclusions regarding the model trained using Thor, DHR, and W/S signals, achieving the highest F1-score (cf. Figure 6). Details in Table 5 show that the improvement in F1-score is due to higher precision on true arousal event identification. The results also reveal that combining Thor, DHR, and Snore signals or Thor, DHR, and Pos signals significantly improves the performance in terms of F1-score. Unlike the other two combinations, using Thor with DHR and Pos signals as input enhances the detection rate of the model (higher recall) while maintaining the same level of precision when compared with the model trained with Thor and DHR signals in Table 4.

### 3.4. Rounds IV and V: Model Trained with Four and Five Input Signals

In the fourth round, we train the model by using all of the Thor, Snore, DHR, and W/S signals. The results show only a slight improvement in the model’s performance compared with the model trained without the Snore signal (cf. Table 5). The model achieves a higher precision score but a lower recall.

In the fifth round, we further add position information to the input; the model’s recall is improved. However, the improvement is not significant compared with the performance obtained in Table 5 using only the Thor, DHR, and W/S signals. Table 6 summarizes the fourth and fifth rounds’ results.

**Figure 7:**
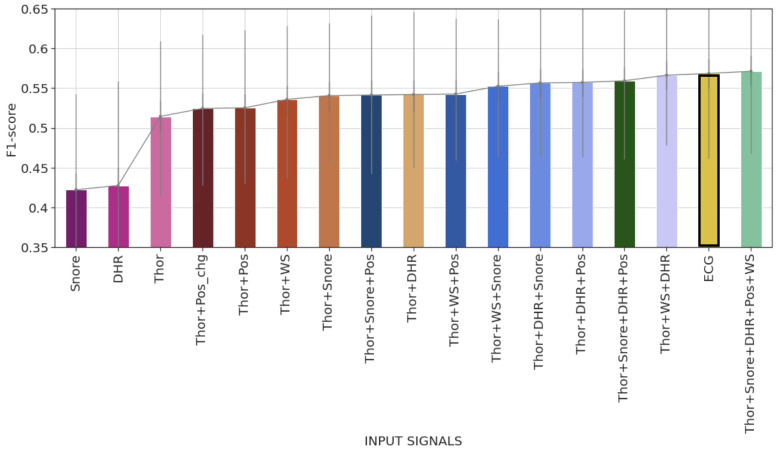
Bar plot of the average recordwise event-based F1-scores of all models trained during the incremental approach. The error bars represent 50% percentile intervals. The gold-colored bar with bold borders represents the mean F1-score of the ECG-based DeepCAD model.

In Figure 7, we summarize the results of the different rounds of the incremental analysis approach. We focus on the per-record F1-score metric, where the F1-score is calculated for each model and record of the test set.

The bar plot in Figure 7 illustrates per-model F1-scores showing the mean and the interquartile interval. It underlines the performance improvement when using the Thor signal compared with DHR and Snore signals, as well as the gradual, continuous enhancement in score when combining the Thor signal with other signals. It is also worth noting that the model trained with Thor+WS+DHR performs similarly to the ECG-based DeepCAD model. Additionally, the model combining all signals slightly improves the F1-score compared with DeepCAD.

### 3.5. Comparison with the State-of-the-Art DeepCAD Model

In this section, we compare the performance of our model when trained using Thor, DHR, and W/S signals as input with the state-of-the-art DeepCAD model proposed by Ao Li et al. [21]. The DeepCAD model is trained using the raw single-lead ECG to detect arousals during sleep. The DeepCAD model’s performance has already been tested on the MESA dataset and has shown promising results.

The comparison results at all levels of evaluation are summarized in Table 7. By only using Thor, DHR, and W/S signals as input, we obtain comparable performance from the DeepCAD model. The model provides higher pointwise AUROC and AUPRC scores, slightly enhancing the pointwise precision and, hence, the F1-score. However, the DeepCAD model performed slightly better according to event-based evaluation (both globally and per record).

In Table 8, we plot the relative confusion matrix comparing the performances of the two models. The off-diagonal values indicate that both models missed almost the same number of events, implying similar detection rates.

We compare the two models’ performances regarding the accuracy of the derived arousal indexes. Our findings reveal a significant Pearson correlation of 0.79 (0.74) between the ground truth ArI and the calculated ArI using the model trained with Thor, DHR, and W/S signals (respectively the DeepCAD model).

**Figure 8:**
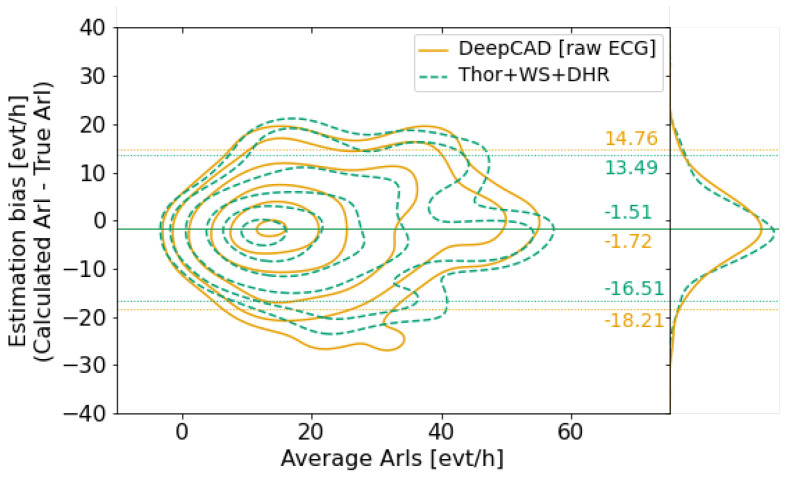
Bland−Altman analysis for calculated ArI versus True ArI. Bland–Altman plots of calculated ArIs using the SoTA DeepCAD model and our DL model trained using Thor+W/S+DHR are superimposed to compare their estimation biases. The Bland−Altman plot shows the estimation bias as a function of the average of the two ArIs. The solid horizontal lines indicate the mean of estimation bias, and the dashed lines show the 95% bounds of estimation bias for each comparison (mean ± 1.96 SD). The bias distributions for the two models are shown on the right. Results show that the two models yield similar results in terms of ArI estimation.

Figure 8 displays Bland–Altman plots that show the difference between the true ArIs and the calculated ArIs of the two models. The plots show that both models slightly underestimate the true ArIs. We can also infer that both models exhibit the same bias distribution with slightly less biased estimates using the Thor, W/S, and DHR signals.

## 4. Discussion

Findings from the first round of our incremental analysis highlighted the significantly better results obtained when the model is trained solely with the thoracic model as input compared with the two other models trained, each with the heart rate and the snore signals. Analyzing changes in the thoracic signal for arousal identification has been scarcely explored in the literature, making it a promising area for further research.

Additionally, our subsequent rounds of analysis demonstrated the effectiveness of a multi-signal approach compared with single-input signal models. By comparing different combinations of input signals, we were able to discern the most important signals for supporting the arousal identification task. Our comparison demonstrated competitive performance with the state-of-the-art DeepCAD model by combining thoracic effort, heart rate, and a wake/sleep indicator signal. The wake/sleep signal used in our study is constructed from the hypnogram. However, our sleep/wake misclassification simulation tests, detailed in Appendix A.5, indicate that even with a reasonable error rate (up to 30%), the W/S signal still improves arousal detection. Identifying new signal combinations for arousal identification holds great promise for future diagnostic device design.

While our findings are encouraging, it is essential to acknowledge that EEG-free arousal identification remains a complex challenge. Further research is required to develop more accurate and reliable models for detecting and characterizing autonomic activations associated with arousal.

One of the key advantages of our data-driven retrospective study is the ability to test a wide range of signal combinations independent of specific testing device capabilities, making it device-agnostic. However, this approach does come with its limitations. First, even when conducted at home, as in the case of MESA, PSG is often performed in a controlled and assisted testing setting that may not accurately replicate real-world unattended home sleep testing conditions using wearable devices. Second, smart wearables may rely on different technologies and sensing techniques, impacting measurement quality and reliability. While signals from smart wearables may not always be less accurate, it is worth noting that the viability of measuring the tested non-EEG signals will depend on the device’s capacity to record or reproduce them. Lastly, while using the MESA dataset allows us to train and evaluate models on large and diverse annotated data, the results may potentially be biased toward specific characteristics of the population targeted by MESA, such as the participants’ age, requiring careful consideration in generalizing our findings to other populations. In the future, we could consider stratifying the training data into different groups based on specific population characteristics and then training the model on a smaller set of the overall training set within each group. This approach would allow for the analysis of how certain population traits affect the model’s performance, providing a more detailed understanding of the generalizability of the study results.

## 5. Conclusions

In conclusion, our study explores the potential of using easily measurable physiological signals to detect arousal events during home sleep testing when EEGs are unavailable. Through a data-driven retrospective analysis utilizing data from MESA and simulating a limited-channel setting from PSG, we found that the thoracic effort signal was the most effective for arousal identification when compared with heart rate and simulated snoring signals. Furthermore, our results showed that continuously combining thoracic effort signals with other easy-to-measure signals led to an improved model performance, highlighting the effectiveness of a multi-signal approach. Our model, trained with the thoracic effort signal, heart rate, and a binary sleep/wake signal, performed similarly to the state-of-the-art ECG-based DeepCAD model. To the best of our knowledge, this combination of input signals has not yet been explored for the EEG-free arousal identification task, even though some existing portable HST devices can easily acquire these signals. Moreover, the comparison results of our study shed light on the most valuable signals for arousal identification. Those results encourage further research to improve the techniques for acquiring and sensing these signals. Finally, we emphasize that additional research is still needed to enhance the model’s ability to detect autonomic activations associated with arousals more accurately and reduce false detections. This will be the focus of our future research.

## Figures and Tables

**Table 1:** The table shows the selected physiological signals as candidate inputs for our arousal detection model and their sources in the MESA dataset.

Signal	Source in MESA Dataset
HR	derived from ECG
Thor	measured by RIP belt
Snore	measured as breath vibrations from cannula
W/S	extracted from the hypnogram
Pos	estimated from the position sensor
Pos_chg	derived from the position signal

**Table 2:** Comparison of the model’s performances when trained using the heart rate (DHR), the thoracic effort (Thor), or the nose cannula vibration snoring (Snore) signal as input. The table summarizes pointwise, event-based and recordwise evaluation results. The results show that the model performs significantly better when trained using the Thor signal as input than the DHR or the Snore signal.

Signals	Thor	DHR	Snore
Pointwise Evaluation			
F1-score	**51.22**	44.23	43.31
Precision	**47.94**	40.95	40.11
Recall	**54.99**	48.07	47.08
AUPRC	**0.54**	0.44	0.42
AUROC	**0.92**	0.88	0.87
Event-based Evaluation			
F1-score	**56.29**	48.67	48.36
Precision	**56.08**	47.56	50.11
Recall	**56.50**	49.82	46.72
Recordwise Evaluation: *mean (SD)*			
F1-score	51.45 ** (15.06)	42.75 (18.05)	42.21 (17.00)
Precision	56.92 ** (16.10)	49.73 (17.60)	48.29 (16.46)
Recall	50.46 ** (17.67)	43.52 (22.72)	41.94 (20.32)

** indicates that the difference in the evaluation score compared with all other trained models is statistically significant (p<0.05). Top performance is marked as bold for each metric.

**Table 3:** Relative confusion matrices comparing the model performance using the Thor signal with the performance using the DHR signal (left) and the Snore signal (right). They show that the Thor signal allows for detecting more arousal events, but 10% of true events were missed when using it but detected with the Snore or the DHR signal. Thus, combining the Thor signal with other physiological signals may increase the arousal detection rate.

		Thor				Thor	
		**Detected**	**Not Detected**			**Detected**	**Not Detected**
**DHR**	**Detected**	11,180	3150	**Snore**	**Detected**	10,986	3189
	**Not detected**	5519	11,938		**Not detected**	5713	11,899

**Table 4:** Comparison of model’s performances when combining the Thor signal with one of the DHR, Pos, Pos_chg, Snore, and W/S signals. The table reports pointwise, event-based, and recordwise evaluation results. The results show that including the DHR, Snore, or W/S signals to the input improves performance in terms of F1-score due to a higher capacity to identify arousals (i.e., higher recall).

	Thor+DHR	Thor+Pos	Thor+Pos chg	Thor+Snore	Thor+WS
Pointwise Evaluation					
F1-score	51.37	49.40	49.19	52.72	**53.90**
Precision	46.39	41.62	40.86	49.57	**52.54**
Recall	57.56	60.77	**61.78**	56.30	55.3
AUPRC	0.55	0.54	0.54	0.56	**0.58**
AUROC	0.92	0.92	0.92	0.92	**0.93**
Event-Based Evaluation					
F1-score	**58.38**	56.15	55.82	57.92	57.34
Precision	**58.22**	51.14	49.93	57.87	55.93
Recall	58.53	62.24	**63.29**	57.96	58.82
Recordwise Evaluation: *mean (SD)*					
F1-score	54.21 ** (15.2)	52.53 (14.82)	52.45 (14.78)	54.07 ** (14.9)	53.59 ** (14.6)
Precision	**57.07 (15.97)**	50.15 (16.26)	49.15 (16.38)	56.55 (15.64)	54.57 (15.53)
Recall	56.15 ** (19.16)	59.38 ** (17.47)	60.58 ** (16.70)	55.89 ** (18.25)	56.66 ** (18.18)

** indicates that the difference in the evaluation score compared with the model trained with only the Thor signal is statistically significant (p<0.05). Top performance is marked as bold for each metric.

**Table 5:** Comparison of the model’s performances when the Thor signal is combined with two signals from DHR, Pos, Snore and W/S signals. The table reports pointwise, event- based and recordwise evaluation results. The results show that the model trained with Thor, DHR, and W/S signals achieves the best F1 and precision scores at different evaluation levels. It provides almost the same recall as the previous round results when Thor+DHR or Thor+W/S signals are considered, as shown in Table 4.

	Thor+	Thor+	Thor+	Thor+	Thor+	Thor+
**Signals**	**WS+DHR**	**DHR+Snore**	**DHR+Pos**	**WS+Snore**	**WS+Pos**	**Snore+Pos**
Pointwise Evaluation						
F1-score	**56.38**	52.43	52.18	55.38	54.03	52.83
Precision	**56.65**	47.21	46.00	54.75	53.52	49.07
Recall	56.12	58.94	**60.26**	56.04	54.56	57.21
AUPRC	**0.61**	0.56	0.56	0.60	0.59	0.56
AUROC	**0.94**	0.92	0.93	**0.94**	0.93	0.92
Event-Based Evaluation						
F1-score	**60.71**	59.77	59.64	59.05	58.13	58.12
Precision	**62.93**	59.83	57.73	58.85	58.73	57.44
Recall	58.63	59.71	**61.69**	59.26	57.55	58.82
Recordwise Evaluation: *mean (SD)*						
F1-score	56.64 ** (14.59)	55.67 ** (14.83)	55.73 ** (14.64)	55.25 (14.34)	54.27 (14.48)	54.14 (15.09)
Precision	61.59 **(14.81)	58.54 ** (15.92)	57.14 (16.05)	56.99 (15.78)	56.76 (16.05)	55.85 (16.11)
Recall	56.46 (19.21)	57.62 (19.07)	59.27 ** (18.93)	57.24 (17.52)	55.47 (17.86)	56.69 (18.27)

** indicates that the difference in the evaluation score compared with models trained with Thor+DHR, Thor+Snore, and Thor+W/S as input is statistically significant (p<0.05). Top performance is marked as bold for each metric.

**Table 6:** Comparison of the model’s performances with Thor, DHR, Snore, and W/S input signals and DHR, Snore, W/S, and Pos input signals. The table reports event-based and pointwise evaluation results regarding F1-score, precision, and recall and the pointwise AUROC and AUPRC. Almost no significant improvement is observed when comparing with Table 5 by adding more signals.

Signals	Thor+Snore+DHR+WS+Pos	Thor+Snore+DHR+WS
Pointwise Evaluation		
F1-score	56.44	**56.50**
Precision	55.72	**57.20**
Recall	**57.18**	55.82
AUROC	**0.94**	**0.94**
AUPRC	**0.61**	**0.61**
Event-Based Evaluation		
F1-score	**61.28**	60.84
Precision	63.47	**64.31**
Recall	**59.24**	57.72
Recordwise Evaluation: *mean (SD)*		
F1-score	**57.13 (14.70)**	56.57 (14.73)
Precision	62.08 (15.34)	62.69 ** (15.24)
Recall	**57.06 (18.91)**	55.59 (19.05)

** indicates that the difference in the evaluation score compared with the model trained with Thor, W/S, and DHR as input is statistically significant (p<0.05). Top performance is marked as bold for each metric.

**Table 7:** Comparison of the model’s performances when trained using the Thor, DHR, and W/S signals with the state-of-the-art DeepCAD model’s performance. The table summarizes pointwise, event-based, and recordwise evaluation results. The results show that the model provides competitive performance using signals easily recordable by HST devices.

		DeepCAD
**Signals**	**Thor+WS+DHR**	**(Uses ECG Signal)**
Pointwise Evaluation		
F1-score	**56.38**	54.84
Precision	**56.65**	51.88
Recall	56.12	**58.15**
AUROC	**0.94**	0.93
AUPRC	**0.61**	0.59
Event-Based Evaluation		
F1-score	60.71	**61.28**
Precision	62.93	**63.73**
Recall	58.63	**59.01**
Recordwise Evaluation: *mean (SD)*		
F1-score	56.64 (14.59)	**56.86 (15.53)**
Precision	61.59 (14.81)	62.94 ** (16.17)
Recall	56.46 (19.21)	**56.69 (20.01)**

** indicates that the difference in the evaluation score compared with the model trained with Thor, W/S, and DHR as input is statistically significant (p<0.05).

**Table 8:** Relative confusion matrices comparing the model performance using the Thor+WS+DHR signal with the DeepCAD performance using ECG. Similar detection rates are obtained by the two models.

		Thor+WS+DHR	
		**Detected**	**Not Detected**
**DeepCAD**	**Detected**	15,368	3393
	**Not detected**	3269	9757

**Figure A2:**
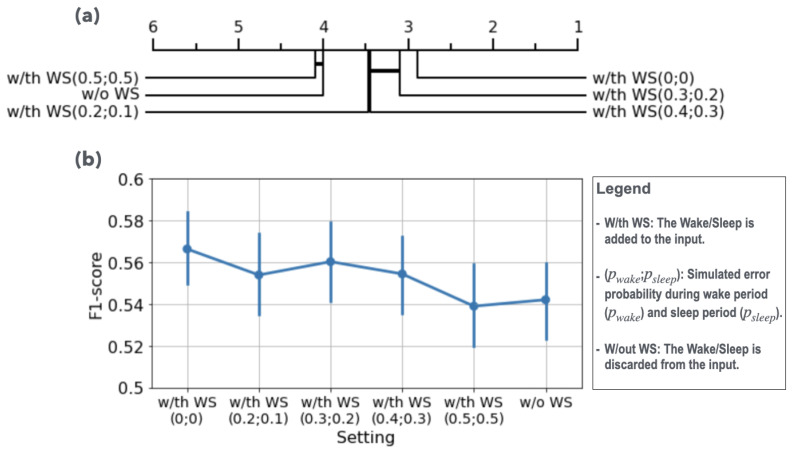
(**a**) Critical difference (CD) diagrams showing the average ranks of models trained with different classification error rates. The lower the rank (farther to the right), the better the performance is. A line in each diagram indicates no significant difference in performance among the models crossed by that particular line in terms of the bootstrapping paired *t*-test with Holm–Bonferroni multiple test correction. (**b**) Error bar plot of the average event-based F1-scores of the different trained models. Error bars represent 50% percentile intervals.

## Data Availability

The data underlying this article are available from the National Sleep Research Resources (NSRR) upon request.

## Abbreviations

The following abbreviations are used in this manuscript:

AASM	American Academy of Sleep Medicine
ArI	arousal index
AUPRC	area under the precision–recall curve
AUROC	area under the receiver operating characteristic curve
DHR	ECG-derived heart rate
ECG	electrocardiogram
EEG	electroencephalogram
EMG	electromyography
HST	home sleep testing
HR	heart rate
HRV	heart rate variability
LSTM	Long Short-Term Memory
MESA	Multi-Ethnic Study of 82 Atherosclerosis
NHLBI	National Heart, Lung, and Blood Institute
NSRR	National Sleep Research Resource
OSAHS	Obstructive Sleep Apnea/Hypopnea Syndrome
Pos	position
${\mathrm{Pos}}_{c}$*h*	position change
PSG	polysomnography
RNN	recurrent neural network
Snore	snoring
Thor	thoracic
WISM	Wearable Intelligent Sleep Monitor
W/S, WS	wake/sleep

## Appendix A

### Appendix A.1. MESA PSG Signal Characteristics

The PSG signals that were recorded during the MESA study and their sampling rates are listed in Table A1.

**Table A1:** PSG signals and their sampling rates.

Channel	Signal	Measurement Sensors	Sampling Frequency (Hz)
EEG1	Encephalogram	EEG gold disk surface electrodes	256
EEG2			
EEG3			
EOG-R	Electrooculogram		
EOG-L			
EMG	Chin electromyogram		
EKG	Electrocardiogram	2 ECG electrodes (+/−) and a ground reference electrode	
Thor	Thoracic effort signal	Compumedics Reuseable Inductive	32
Abdo	Abdominal signal	Belts for Summit IP/Somte Systems	
Flow	Airflow signal	Salter ThermiSense and nasal cannula	
Snore	Snoring signal		
Leg	Leg movement signal	Compumedics Leg Piezo Sensors	
Pos	Position signal	Built-in position sensor attached to the monitoring unit vest	
SpO2	Oxygen saturation signal	Nonin 8000J Flex oximeter finger sensor	1
DHR	Derived HR from ECG		

### Appendix A.2. Exclusion Criteria

The exclusion criteria for this study are detailed in the flowchart in Figure A1. Records with low-quality ECG, DHR, Snore, or Pos signals are discarded from the MESA dataset. Records without annotations for wake/sleep or micro-arousals are also excluded.

### Appendix A.3. MESA Training, Validation, and Test Set Characteristics

Table A2 summarizes the characteristics of the training, validation and test sets.

**Table A2:** MESA training, validation, and test set characteristics.

	Train	Validation	Test
Count	952	104	255
% Female	53.7	51.9	59.2
Recording time *	636.5 ± 78.53	630.83 ± 114.77	637.8 ± 96.36
Age *	69.08 ± 9.05	68.5 ± 8.67	68.67 ± 8.7
AHI *	20.28 ± 18.27	19.88 ± 19.75	18.7 ± 11.8
ArI *	22.53 ± 11.86	22.24 ± 13.31	21.99 ± 15.79

* mean ± SD.

### Appendix A.4. Postprocessing: Number and Percentage of Merged and Discarded Events

The output of the deep learning model is further processed to better align with the AASM guidelines and to smooth the model predictions. First, events that are less than 5 s apart are merged. Second, events lasting less than 3 s are discarded. Table A3 shows the number and percentage of detected arousals that were combined and those that were discarded for each model.

**Table A3:** Number and percentage of merged and discarded arousals per model.

Model	Count of Detected Events	Count of Merged Events (Percentage %)	Count of Discarded Short Events (Percentage %)
**DeepCAD (ECG)**	63,771	558 (0.87)	2006 (3.15)
**Thor**	67,818	1131 (1.67)	2909 (4.29)
**DHR**	69,301	1003 (1.45)	3235 (4.67)
**Snore**	66,231	1922 (2.9)	2977 (4.49)
**Thor+DHR**	66,895	1016 (1.52)	2186 (3.27)
**Thor+Pos**	75,186	1431 (1.9)	3337 (4.44)
**Thor+Pos_chg**	78,093	1906 (2.44)	4159 (5.33)
**Thor+Snore**	67,058	769 (1.15)	2686 (4.0)
**Thor+WS**	69,577	954 (1.37)	3412 (4.9)
**Thor+WS+Snore**	67,468	780 (1.16)	2895 (4.29)
**Thor+WS+DHR**	64,212	684 (1.07)	2131 (3.32)
**Thor+WS+Pos**	66,772	1025 (1.54)	2811 (4.21)
**Thor+DHR+Pos**	69,055	946 (1.37)	2399 (3.47)
**Thor+DHR+Snore**	66,774	941 (1.41)	2361 (3.54)
**Thor+Snore+Pos**	68,131	962 (1.41)	2756 (4.05)
**Thor+Snore+DHR+WS**	63,171	799 (1.26)	2051 (3.25)
**Thor+Snore+DHR+WS+Pos**	64,191	767 (1.19)	1969 (3.07)

### Appendix A.5. Wake/Sleep Misclassification Simulation Tests

In order to assess how the quality of the wake/sleep (W/S) signal affects the model’s performance, we conducted various simulations. We trained the model while introducing a progressively increasing probability of classification errors into the W/S signal during both wake and sleep periods. These error rates ranged from 20% to 50% during wake periods and from 10% to 50% during sleep periods.

The critical difference (CD) diagrams, illustrated in Figure A2a, show the mean ranks of each model under different classification error rates. The ranks of the model trained with no classification error and the model without using the W/S signal are also included. The rankings are calculated based on the event-based F1-score across the test records. Horizontal lines in the diagram indicate no significant difference in performance among the models. Additionally, in Figure A2b, we show the average F1-score for each model, with bars representing the 50th percentile interval. Results show that a moderate classification error in the W/S signal (around 10–30%) leads to a decrease in score but still improves the model’s performance compared with the model trained without a W/S signal. Furthermore, even when a significant error is introduced in the W/S signal (refer to setting with 50% classification error), the model still performs as well as it does without using the W/S signal.
